# Antidepressive and antinociceptive effects of ethanolic extract and fruticuline A from *Salvia lachnostachys* Benth leaves on rodents

**DOI:** 10.1371/journal.pone.0172151

**Published:** 2017-02-21

**Authors:** Joyce Alencar Santos, Ana Claudia Piccinelli, Maira Dante Formagio, Cristhian Santos Oliveira, Elide Pereira dos Santos, Maria Élida Alves Stefanello, Ubirajara Lanza Junior, Rodrigo Juliano Oliveira, Mário Mateus Sugizaki, Cândida Aparecida Leite Kassuya

**Affiliations:** 1 Federal University of Grande Dourados, College Exact Sciences and Technology, Dourados, Mato Grosso do Sul, Brazil; 2 Federal University of Grande Dourados, College of Health Science, Dourados, MS, Brazil; 3 Federal University of Paraná, Department of Chemistry, Curitiba, PR, Brazil; 4 Federal University of Paraná, Department of Botany, Curitiba, PR, Brazil; 5 Federal University of Mato Grosso do Sul, College of Health Science, Campo Grande, MS, Brazil; 6 Federal University of Mato Grosso, College of Health Science, Sinop, MT, Brazil; University of Würzburg, GERMANY

## Abstract

**Objectives:**

This study investigated the antidepressant and antinociceptive effects of ethanolic extract (SLEE) and pure fruticuline A obtained from *Salvia lachnostachys* leaves on rats and mice.

**Methods:**

In this study, SLEE (100 mg/kg, p.o. route) was evaluated for its effects on spared nerve injury (SNI) in rats. The animals were submitted to mechanical sensitivity, forced swim (FST) and cold sensitivity tests 10 and 15 days after surgery. SLEE (100 mg/kg, p.o.) and fruticuline A (3 mg/kg, p.o.) were also evaluated with respect to nociceptive behavior induced by formalin. In addition, clonidine-induced depressive-like behavior was also analyzed.

**Results:**

The oral administration of SLEE for up to 15 days and the subcutaneous injection of 10 mg/kg of ketamine (positive control) significantly inhibited SNI-induced mechanical hyperalgesia and decreased immobility in the FST. On the 15th day of oral treatment, SLEE prevented the SNI-induced increase in cold sensitivity. In the formalin test, SLEE and fruticuline A significantly reduced the frequency of paw licking during the first and second phases and decreased the formation of edema. In locomotor analysis (open field test without clonidine treatment), SLEE and fruticuline A did not alter the response. SLEE and fruticuline A significantly attenuated clonidine-induced suppression of spontaneous locomotor activity (squares invaded and licking) and emotionality (grooming and freezing) compared with controls, similar to the naive group.

**Conclusion:**

SLEE exhibits antihyperalgesic, antidepressant, and antinociceptive effects, and fruticuline A appears to be at least partly responsible for the effects of SLEE. Together, these results demonstrate the antidepressive effects of SLEE and fruticuline A and indicate that both derivatives obtained from *S*. *lachnostachys* act against spontaneous neuropathic pain.

## Introduction

The *Salvia* genus (Lamiaceae) comprises approximately 1000 species distributed worldwide [1]. The antidepressive and anxiolytic effects of *S*. *divinorum* and *S*. *elegans* have been reported [2–5]. Studies have demonstrated anti-inflammatory and antinociceptive effects in different *Salvia* species [4–6].

*Salvia lachnostachys* Benth is an herb that is native to Brazil and whose ethanolic extract from leaves (SLEE) has been studied. Triterpenes and diterpenes, including fruticuline A, were previously isolated from SLEE using chromagraphic techniques and identified by ^1^H and ^13^C NMR [7]. SLEE and fruticuline A exhibited anti-inflammatory (paw edema and pleurisy induced by carrageenan injection) and antihyperalgesic inflammatory effects. Fruticuline A dose tested was evaluated in the yield in SLEE (3%) and with this dose relationship, the compound also prevented mechanical hyperalgesia, inhibiting tumor necrosis factor (TNF) [6]. These results prompted us to investigate the effects of SLEE in other types of pain, such as neuropathic pain-induced hyperalgesia (not only inflammatory pain), as well as related problems, such as depression.

The etiology of depression is uncertain, but several pharmacological treatments (reserpine, clonidine) and conditions lead to depression of the central nervous system (CNS), such as neuropathic pain and infection. Neuropathic pain is commonly associated with cognitive and emotional comorbidities, such as depression, and the mechanism appears to involve central sensitization and structural changes in the brain [8]. Neuropathic pain is a type of chronic pain that develops from a disease or direct damage to the somatosensory nervous system, which contrasts with the normal plasticity in nociceptive pain [9]. This persistent type of pain is characterized by hyperalgesia, hyperpathia and allodynia. Neuropathic pain affects millions of people worldwide and is considered the most difficult type of chronic pain to treat, often disturbing patients’ quality of life and impacting society [10, 11]. Spared nerve injury (SNI) experimental models induce chronic mechanical and thermal hypersensitivity, with a rapid initiation and prolonged effect. This model is considered a good neuropathic pain model in animals [12].

In addition, infection and inflammation appear to induce symptoms of CNS depression and associated pains. However, it remains unclear whether the depressive mental state is caused by pain or directly induced by infection. Due to this complex interaction, there are no effective therapeutic strategies to control both pain and behavioral and cognitive changes based on anticonvulsants and opioids, which are normally ineffective [13].

We investigated the antihyperalgesic, antidepressant and antinociceptive effects of SLEE using SNI as an experimental model for hyperalgesia and depression. SLEE and fruticuline A were assayed for formalin and clonidine-induced depression.

## Materials and methods

### Plant material-extraction and isolation of fruticuline A

Leaves of *S*. *lachnostachys* Benth were collected in Curitiba, Paraná, Brazil (25°30'44.6''S, 49°18'7.13''W). A voucher was deposited in the Herbarium of the Federal University of Paraná (UPCB, Santos1251). *S*. *lachnostachys* is not an endangered or protected species, and authorization is not required for its collection. The plant was collected from urban, private land that had been cleared for building purposes. The owner gave permission to remove the plants. Dried and powdered leaves (415.3 g) were extracted at room temperature with hexane followed by ethanol. The solvents were removed under reduced pressure to give the crude extracts in hexane and ethanol (SLEE). The hexane extract was not studied. SLEE (45.0 g) was fractionated by silica gel column chromatography (CC) and eluted with solvents in order of increasing polarity (hexane, CH_2_Cl_2_, acetone and MeOH). The fraction eluted with hexane (1.8 g) was submitted to further CC and eluted with several solvents, yielding 14 fractions. Fruticuline A (63 mg) was obtained from fraction 6 (200 mg) after preparative TLC in toluene or benzene:CH_2_Cl_2_ 8:2. Fruticuline A was identified by NMR analysis and literature comparison [7, 14, 15].

### Animals

Experiments were conducted using male *Wistar* rats (250–350 g) and male *Swiss* mice (20–30 g). The animals were maintained on a 12-h light/12-h dark cycle at a constant temperature of 22±1°C with controlled humidity (60–80%). Food and water were provided *ad libitum*. The animals were acclimatized to the experimentation room for at least 2 h before testing and were used only once throughout the experiments. All of the experimental procedures were approved by the Ethics Committee on Laboratory Animals of UFGD (No. 005/2014).

### Spared Nerve Injury model (SNI) and treatments

Male Wister rats received an intraperitoneal injection (i.p.) of ketamine hydrochloride (Vetbrands, Brazil) (60 mg/kg) and xylazine (Syntec, Brazil)(10 mg/kg) for anesthesia. A total of 8 rats were used as sham animals, in which the sciatic nerve was exposed but not manipulated. In 40 operated SNI animals, the right sciatic nerve was exposed, and the tibial and common peroneal nerves were tied with 2 knots using 6.0 silk after they were cut [12, 16]. After surgery, the muscle and skin were sutured.

#### SNI-treated groups

All treatments were performed by oral route (p.o.) with a daily single dose for 15 days for the SLEE (100 mg/kg), control (saline 0.9%) and sham (saline 0.9%) groups and by i.p. for the ketamine group (1 mg/kg, positive control). The dose of SLEE was determined based on a previous study by our group [6].

Tests of mechanical and cold sensitivities and a forced swim test (FST) were conducted on all operated rats 10 and 15 days after SNI procedures.

Mechanical sensitivity (nociceptive threshold (g)) should be changed to was measured in the right hind paw using an electronic version of the von Frey test (Insight, Brazil) [12]. Vocalization or withdraw indicated that the rats felt pain [17]. Before testing, the rats were left in the test cages for 15 to 20 min for ambient adaptation. When testing SNI animals, special care was taken to stimulate the lateral plantar surface[12].

Cold hyperalgesia was measured by the acetone drop test [12]. A syringe was adapted and used to drop 30 μL of acetone (Próquímicos, Brazil) on the paw, and the duration (in seconds) of paw withdrawal was analyzed. Minimal and maximal cut-offs were assigned at 0.5 and 20 seconds.

One day before SNI surgery, each animal underwent a FST [18]. Rats were individually forced to swim in an open cylindrical container (diameter 60 cm, height 100 cm) containing 30 cm of water at 25±1°C; the total duration of immobility during the 5-min test was observed.

### Open field test

Fifty minutes after p.o. with SLEE (100 mg/kg), fruticuline A (3 mg/kg) or vehicle (saline and tween 80, 0.1%), the mice were placed individually in the center of the arena, and their locomotor activity was quantified for 5 min. The number of "squares" invaded (ambulation) in the center and the periphery of the arena were analyzed. Ambulation was used to evaluate the horizontal movement/exploratory activity. Reduced locomotion is related to anxious behavior.

### Formalin-induced nociceptive behavior and treatments

One hour after oral treatment, nociception was induced by the administration of 2.5% formalin in the paw. The animals were individually placed on transparent observation platforms (under inverted funnel with a glass mirror on the back) for a period of adaptation of at least 20 min. Each mouse received a formalin solution (20 μL, 2.5% formaldehyde (Proquímicos, Brazil) 0.92%) by intraplantar route in the right hind paw and 20 μL of saline into the left hind paw. And a naive group that received 20 μL of saline intraplantar route in the right hind paw. Immediately after formalin injection, the mice were placed on observation decks. The nociceptive behavior was determined as the time (seconds) that the animal continued to lick, bite or raise the paw injected with formalin and was observed for 30 min. This model is biphasic, thus allowing the assessment of pain sensitivity in two phases. The first phase occurs during the first 5 min after formalin injection, and the second phase is 15–30 min after formalin administration. Edema was measured (in mL) using a plethysmometer (PANLAB Harvard, Spain) as differences between the left and right paws after 2 h [19].

### Model of depression by clonidine

Animals were classified into two main groups: naive (n = 6) and depressed (n = 18). Depression was induced by the i.p. of clonidine (Cristália, Brazil) (0.8 mg/kg) daily for 7 successive days [20]. The naive group received the i.p. of saline for the same duration. Thereafter, depressed mice were subclassified into 3 groups, each consisting of 6 rats. From the fifth to seventh days, animals were orally treated with the following: saline and 1% tween 80 (naive and control groups), fruticuline A (3 mg/kg) and SLEE (100 mg/kg). The open field test was performed 24 h after the last treatment. One hour later, blood serum was obtained for subsequent analysis, and animals were sacrificed.

### Statistical analysis

Data are presented as the mean±standard error of mean (SEM). Differences between groups were evaluated by analyses of variance (one-way ANOVA) followed by the Newman-Keuls test. The number of animals per group is indicated in the legends. Significant differences were considered significant at *P*<0.05. Asterisks (*) denote significant differences compared with the vehicle-treated group.

## Results

**Fig 1:**
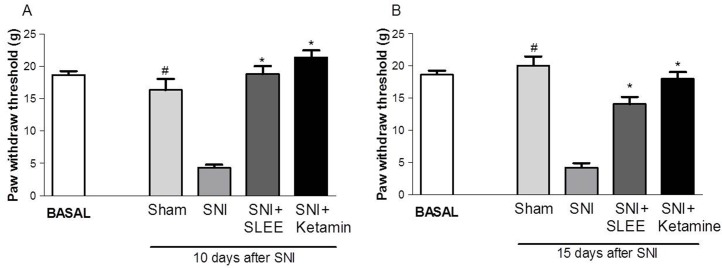
Effect of oral administration of SLEE on SNI-induced increases in mechanical sensitivity (paw withdraw threshold) in rats. A and B show the values 10 and 15 days after SNI, respectively. The bars represent the basal value (before surgery) and post-surgery Sham, SNI, SNI+SLEE (100 mg/kg, p.o.) SNI + ketamine (10 mg/kg, i.p.). Animals were treated once a day for 15 days after the SNI and sham procedures. Bars express the mean±SEM (n = 8) comparing the SNI vs. treated and sham groups. *P<0.001 or ^#^*P*<0.001.

At 10 and 15 days, the sensitivity to a mechanical stimulus increased in the SNI group compared with the control group (Fig 1A and 1B). Furthermore, 100 mg/kg of SLEE administered once daily for 15 days after surgery prevented 100% (*P*<0.001) of the SNI-induced increase in sensitivity to mechanical stimulus when measured post-surgery. Ketamine inhibited the mechanical hyperalgesia induced by SNI on all days tested.

**Fig 2:**
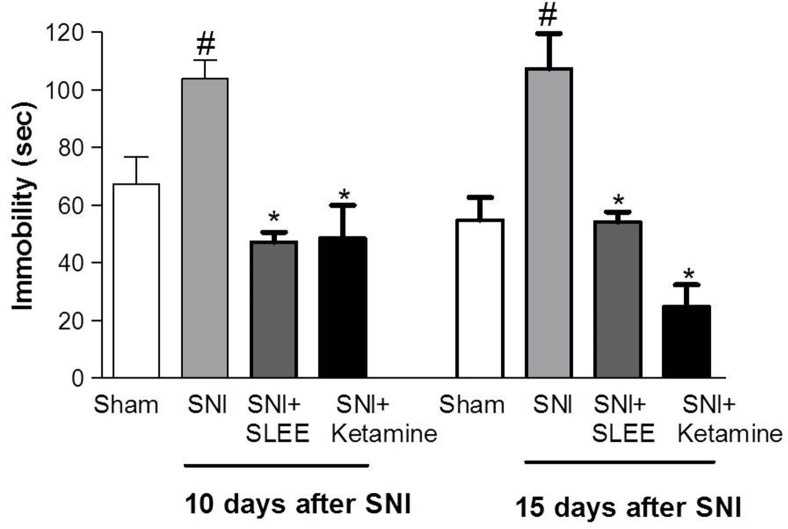
Effect of oral administration of SLEE on SNI-induced increases in immobility in rats during the forced swim test. The groups received Sham, SNI, SNI+SLEE (100 mg/kg, p.o.) or SNI+ketamine (10 mg/kg, i.p.) treatments. Bars represent the time of immobility on the 10^th^ and 15^th^ days after SNI. Bars express the mean±SEM (n = 8) comparing the SNI vs. treated (*P<0.05) or sham (^#^*P*<0.001) groups.

The immobility of the SNI group increased approximately by 37 and 49% 10 and 15 days post-surgery, respectively, compared with the sham group. SLEE decreased immobility during the FST on both days, with inhibitions of 55% (day 10) and 50% (day 15). Ketamine significantly reduced the immobility induced by SNI (53% 10 days and 77% 15 days after SNI surgery) (Fig 2).

**Fig 3:**
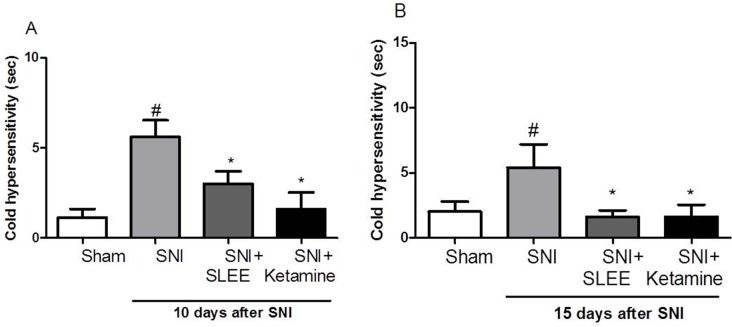
Effect of the oral administration of SLEE on SNI-induced increased cold sensitivity in rats. The groups received Sham, SNI, SNI+SLEE (100 mg/kg, p.o.), or SNI+ketamine (10 mg/kg, i.p.). In (A, B), bars represent the time of the response to cold stimulus on the 10^th^ day after SNI. In (B), bars represent the cold sensitivity on the 15^th^ day after SNI. The bars express the mean±SEM (n = 8) comparing the SNI vs. treated (*P<0.05) or sham (^#^*P*<0.05) groups.

SLEE also prevented cold sensitivity in the SNI model as evaluated 10 and 15 days after surgery. Fig 3 indicates that cold sensitivity was detected and that increased approximately 7-fold. In addition, the SNI group differed significantly from the sham and basal groups (*P<*0.05) (Fig 3A). On the 15^th^ day of daily oral treatment with SLEE, the SNI-induced increase in cold sensitivity significantly decreased (Fig 3B).

Oral administration of SLEE (925.2±23.5) and fruticuline A (1131±43.7) did not cause statistically significant differences in analyses of locomotor activity during the open field test compared with the control group (1008±46.37).

**Fig 4:**
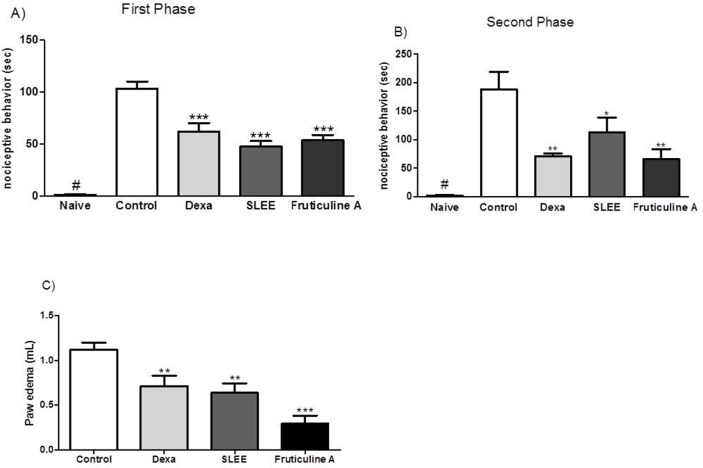
Effect of the oral administration of SLEE and fruticuline A on formalin-induced nociceptive behavior and the neurogenic edema test. In (A, B), the time that the animals spent licking or biting during the first phase (0–5 min) and the second phase (15–30 min) is reported. In (C), neurogenic edema is presented. Bars indicate animals that received control or naive (saline 0.9%), dexamethasone (1 mg/kg), SLEE (100 mg/kg, p.o.) or fruticuline A (3 mg/kg, p.o.). Bars express the mean±SEM (n = 6) comparing the vehicle vs. treated groups (*P<0.05; **P<0.01 and ***P<0.001).

The naive group that received only intraplantar injection with 20 μL of saline did not exihibit nociceptive behavior but formalin solution, in the control group, increased significantly the licking behavior. Nociception induced by the administration of formalin in the paw was evaluated at two stages. The first stage was 0–5 min, and the second stage was 15–30 min after the induction of nociception. Compared with the controls, nociception in the SLEE and fruticuline A groups decreased by 54 and 48% in the first phase and by 40 and 65% in the second phase (Fig 4A), respectively. SLEE and fruticuline A decreased neurogenic edema formation through induction in a formalin model, and these effects were evaluated 50 min after the administration of formalin. Fig 4B indicates that decreased paw edema was detected in the groups treated with SLEE (43%) and fruticuline A (69%) compared with the control group.

**Fig 5:**
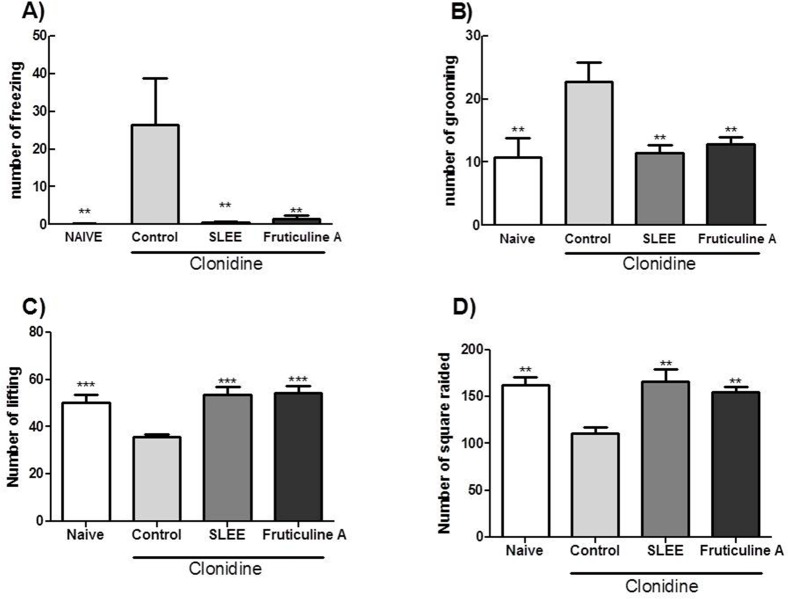
Effect of fruticuline A and SLEE during the open field test in clonidine-induced depressive mice. The animals were treated with 8 mg/kg (i.p.) of clonidine for 7 days, and the naive group received saline (i.p.). From the fifth to seventh days, animals received an p.o. of saline (naive and control), SLEE (100 mg/kg) and fruticuline A (3 mg/kg.). On the eighth day, the number of freezing (A) and grooming (B) animals were assessed, indicating emotionality. Locomotor activity was evaluated by the number of licking animals (C) and squares invaded by animals (D). The bars express the mean±SEM (n = 6) compared with control (**P<0.01 and ***P<0.001).

The model of depression induced by clonidine administration was assessed by the open field test. A significant reduction in the number of freezing animals treated with fruticuline A and SLEE was observed. In addition, the number of grooming mice was also reduced (Fig 5A and 5B), indicating that the emotional behavior of the animal was restored. These results did not differ from the naive group (not depressive). Locomotor activity also exhibited a statistically significant difference compared with the control group, as measured by the number of invaded squares and licking; these results were similar to the naive group (Fig 5C and 5D).

## Discussion

In the present study, the main findings include the antidepressive and antinociceptive effects of SLEE and pure fruticuline A, which was isolated from SLEE. This compound is a *nor*-abietane diterpenoid with antimicrobial [15], anti-inflammatory [6], and cytotoxic effects against the activity of human cancer cell lines [7]. The relationship between fruticuline A and SLEE is that crude extracts contain about 3% of this compound and all tests made with fruticuline A followed this proportion leading similar efficacy with both products from *S*. *lachnostachys*.

The antidepressive activity of SLEE was demonstrated in SNI-induced depressive rats and clonidine-induced depressive mice. In the present study, SLEE exhibited effective antinociceptive effects against spontaneous pain (formalin test), stimulus-evoked pain, and neuropathic pain (SNI model). Previous results from our group led to the isolation and demonstration of anti-inflammatory action of fruticuline A from SLEE against mechanically-induced inflammatory pain [6]. The results indicate that this compound inhibits nociception in response to formalin-induced stimulus-evoked pain and depressive effects induced by clonidine. Together, these results demonstrate the antidepressive effects of SLEE and fruticuline A and indicate that both products from *S*. *lachnostachys* are effective against spontaneous and neuropathic pain.

Neuropathic pain is a type of chronic pain that develops from a disease or from direct damage to the somatosensory nervous system, in contrast to the normal plasticity in nociceptive pain [9–11]. The SNI experimental model induces chronic mechanical and thermal hypersensitivity with a rapid initiation and prolonged effect, and this model is considered a good neuropathic pain model in animals [12].

In the present investigation, the administration of SLEE negatively modulated neuropathic pain (mechanical hyperalgesia, cold sensibility) induced by SNI. Based on our data and with support from the literature, SLEE may have an ameliorative effect in SNI-induced painful peripheral neuropathy by virtue of its multiple effects. Its anti-inflammatory, antinociceptive and antidepressive effects were manifested in terms of alleviating SNI-induced behavioral alterations.

The consequences of neuropathic pain are depression, anxiety and anhedonia. These symptoms are also reproduced in animal models of peripheral nerve injury. Monoamine neurotransmission dysfunction in the CNS has been proposed in an attempt to base these consequences on underlying depressive disorders in neuropathic pain [21]. In animal models, pain is assessed by sensory hypersensitivity, whereas depression can be measured by FST, sucrose preference [22], tail suspension or open field tests [23]. The FST is commonly used to assess depression in animal models and screen for new antidepressant agents [18]. Ketamine has fast antidepressant effects in rodents because it can rapidly increase synaptic proteins and the number and function of synaptic connections in the prefrontal cortex, as well as reduce synaptic deficits [24].

Porsolt et al. (1977) assessed antidepressants standards and noted that these standards reduced immobility in the FST, indicating an antidepressant effect. In the present study, the administration of 10 mg/kg of ketamine significantly decreased the immobility time of rats undergoing the FST. The quality of life is severely impaired, and treatment is necessary with conventional drugs, such as painkillers and antidepressants, as well as alternative natural substances from medicinal plants and/or herbal remedies [25]. To contribute to the advancement of patho-physiological studies of these conditions using biologically active plant substances, previous work in our laboratory demonstrated the analgesic and anti-inflammatory effects of ethanolic extract of *S*. *lachnostachys* and fruticuline A (a biologically active plant component that comprises the majority of the extract) in a paw edema model in mice [6]. This result is consistent with previous studies [24] and confirms that the antidepressant activity of SLEE inhibits these models.

Acetone produces a distinct cooling sensation as it evaporates. Normal rodents do not respond to this stimulus or exhibit a minimal response (in amplitude and duration), whereas sensitized animals will almost always respond with an exaggerated response [26]. Our results indicate insensitivity in sham animals after surgery. The animals were sensitive to cold, and this sensitivity decreased after oral treatment with SLEE.

In addition, the antidepressive effect of these natural biologically active compounds (at the doses described above) was assayed in a model of monoamine-deficit dependent depression induced by clonidine [20]. Clonidine was previously prescribed as an antihypertensive. Clonidine is an adrenergic agonist of the direct action of a presynaptic α-adrenergic receptor located in the CNS that is involved in the negative feedback of catecholamine release. However, new clinical uses of clonidine include opiate detoxification; sleep hyperhidrosis; antagonism of the side effects of psychostimulants, such as methylphenidate and amphetamines; and the treatment of various types of neuropathic pain at low doses (1 μg/kg) compared with the dose used to induce CNS depression in experimental models [27]. In this study, the oral administration of SLEE and fruticuline A prevented the development of CNS depression induced by clonidine compared with the respective group controls. There are several reports in the literature regarding the antidepressant activity of plant extracts and/or active substances isolated from plants, such as Salvia divinorum [2] and *Canavalia brasiliensis* [28]. However, specific studies on the bioactive components present in the ethanolic extract of *S*. *lachnostachys* describing their biological effects on the CNS are lacking. Plants containing terpenoids have been used in folk medicine as sedatives, tranquilizers, antidepressants and anticonvulsants [29, 30]. Thus, there is a need for more scientific studies on the structure-activity relationship of compounds found in *S*. *lachnostachys* with a focus on their biological properties. Studies should also address how prototypes can offer more effective pharmacology and ensure the pharmacotherapy of affective changes, including depression of the CNS, observed in the presence of neuropathic pain.

The evaluation of the open field test 50 min after a single oral dose of SLEE and Fruticuline A revealed that the extract does not change the behavior of the animals. In this experiment, the animals travelled and explored the environment of the open field in a manner that was similar to the control group, which received the vehicle.

The intraplantar injection of formalin is a well-established model of persistent pain characterized by a transient, biphasic pattern of pain behavior that comprises two stages of painful sensitivity. In the first stage, neurogenic pain is caused by the acute activation of C and Aδ fibers by neuropeptides, such as substance P [31]. The second stage is characterized as inflammatory pain and is related to the release of chemical mediators, such as histamine, serotonin, bradykinin, prostaglandins and excitatory amino acids. These mediators can be inhibited by painkillers and ant-iinflammatory drugs. SLEE and fruticuline A decreased painful sensitivity compared with the control group. Reductions of 54% and 48%, respectively, were noted in the first stage and 40% and 65%, respectively, in the second stage. These results indicate that the extract and its compound fruticuline A are effective against neurogenic pain. The inflammation caused by formalin, as characterized by paw edema, was reduced by SLEE (43%). SLEE produced results similar to dexamethasone, an anti-inflammatory drug. Fruticuline A promoted an even greater reduction in paw edema (69%). Fruticuline A alone was more efficient than SLEE, suggesting that it may be one of the substances responsible for the antinociceptive effects. Consistent with our results, SLEE also significantly reduced paw edema caused by carrageenan, and fruticuline A produced an effect statistically similar to that of dexamethasone [6], which has anti-inflammatory activity, showing a reduction in the formation of paw edema similar to that reported in the literature. The drug showed a 63% reduction in effective nociception in the second stage, while the reduction in the first phase of nociception was close to 37%. As previously reported, the mechanism of this drug acts during the second stage [32].

The results from our group revealed that fruticuline A is responsible for the anti-inflammatory effect of SLEE [6] and is possibly that Fruticuline A also be important for antihyperalgesic and antinociceptive effects of SLEE. Fruticuline A inhibited only the TNF, but not DOPA, effects in mice paw [6] suggesting that the main mechanisms of Fruticuline A in inflammation and pain effects is by inhibition of TNF activation.

## Conclusion

This study revealed that ethanol extract from leaves of *S*. *lachnostachys* (SLEE) has antinociceptive effects in a formalin model, antihyperalgesic effects against mechanical stimulus- and cold stimulus-evoked pain in a neuropathic pain model, and antidepressive effects in a neuropathic pain and clonidine-induced depression model in rodents. Fruticuline A reduces the nociception induced by formalin and depressive actions induced by clonidine. Fruticuline A contribute (at least partially) to the effects of SLEE. Fruticuline A had antinociceptive and also antiedematogenic effects.
